# Accelerated DNA methylation age and the use of antihypertensive medication among older adults

**DOI:** 10.18632/aging.101626

**Published:** 2018-11-10

**Authors:** Xu Gao, Elena Colicino, Jincheng Shen, Allan C. Just, Jamaji C. Nwanaji-Enwerem, Brent Coull, Xihong Lin, Pantel Vokonas, Yinan Zheng, Lifang Hou, Joel Schwartz, Andrea A. Baccarelli

**Affiliations:** 1 Department of Environmental Health Sciences, Mailman School of Public Health, Columbia University, New York, NY 10032, USA; 2 Department of Environmental Medicine and Public Health, Icahn School of Medicine at Mount Sinai, New York, NY 10029, USA; 3 Department of Population Health Sciences, University of Utah, School of Medicine, Salt Lake City, UT 84132, USA; 4 Department of Environmental Health, Harvard T.H. Chan School of Public Health, Boston, MA 02115, USA; 5 Department of Biostatistics, Harvard T.H. Chan School of Public Health, Boston, MA02115, USA; 6 Veterans Affairs Normative Aging Study, Veterans Affairs Boston Healthcare System, Department of Medicine, Boston University School of Medicine, Boston, MA 02118, USA; 7 Department of Preventive Medicine, Feinberg School of Medicine, Northwestern University, Chicago, IL 60611, USA

**Keywords:** DNA methylation age, antihypertensive medication, aging, epigenetic epidemiology, hypertension

## Abstract

The discrepancy of DNA methylation age (DNAmAge) with chronological age (termed as age acceleration, AA) has been identified to be associated with many aging-related health outcomes including hypertension. Since taking antihypertensive medication (AHM) could prevent aging-related diseases caused by hypertension, we hypothesized that using AHM could also reduce the AA. We examined this hypothesis among 546 males aged 55–85 years by exploring the associations of AHM use with AA and its change rate (Δ_AA_) in two visits with a median follow-up of 3.86 years. Horvath DNAmAge was derived from DNA methylation profiles measured by Illumina HumanMethylation450 BeadChip and information on AHM use was collected by physician interview. A general decreasing pattern of AA was observed between the two visits. After the fully adjusting for potential covariates including hypertension, any AHM use showed a cross-sectional significant association with higher AA at each visit, as well as a longitudinal association with increased Δ_AA_ between visits. Particularly, relative to participants who never took any AHM, individuals with continuous AHM use had a higher Δ_AA_ of 0.6 year/chronological year. This finding underlines that DNAmAge and AA may not be able to capture the preventive effects of AHMs that reduce cardiovascular risks and mortality.

## Introduction

DNA methylation, a major form of epigenetic modification, is known to play an important role in aging and the development of age-related health outcomes [1,2]. Recently, a DNA methylation-based biological age predictor, “DNA methylation age (DNAmAge)”, has been established and found to be highly associated with chronological age [3]. The discrepancy between this epigenetic-based indicator and the chronological age has been termed age acceleration (AA), which was found to be heritable and has been used as an index of accelerated biological aging. Follow-up investigations have linked AA to lifestyle factors, environmental hazards, stressful life events, as well as all-cause mortality [4–13].

Several aging-related factors, including inflammation, neurohormonal disorder and endothelial dysfunction, have been found to play key mechanistic roles in the development of hypertension [14–16], the most common long-term medical condition among older adults that could lead to various forms of age-related health outcomes, such as cardiovascular diseases (CVD), kidney failure and dementia [17]. Relationships of hypertension and blood pressure with biological aging have also been studied since the introduction of DNAmAge. In 2016, Horvath et al. found that people with hypertension had a higher AA (0.5 – 1.2 years) in comparison to controls in the Bogalusa Heart study [7] and a more recent study from Quach et al. showed that elevated blood pressure was also correlated with higher extrinsic and intrinsic DNAmAge [10].

The use of antihypertension medication (AHM) reduces the risk of adverse age-related health outcomes caused by hypertension. Specifically, observational studies, clinical trials, and systematic reviews mostly suggested that effective antihypertensive therapy greatly reduces the risk of CVD in patients with hypertension [18,19], and may also be associated with a decreased risk of cognitive decline and incident dementia [20]. As DNA methylation is a durable and reversible modification, we hypothesized that the use of AHMs might also be able to influence the biological aging reflected by the epigenetic AA. Therefore, we assessed the associations of AHM use with AA and further determined whether the change of AHM use could modify the change rate of AA (Δ_AA_) during a median follow-up of 3.86 years. This investigation was carried out in the Normative Aging Study (NAS), which is an all-male longitudinal study of a cohort of older veterans living in the Greater Boston area.

## RESULTS

### Participant characteristics

Characteristics of the 546 participants at each visit are shown in Table 1. Overall, the average age at first and second visits was 72 and 75 years, respectively. More than 60% of the participants were former smokers and less than 5% were current smokers, and the majority of participants were overweight or obese, consumed no or low amounts of alcohol, reported low physical activity and had less than 16 years of education. During a median follow-up of 3.86 years, the prevalence of hypertension increased from about 68% to 75%, stroke from nearly 6.0% to 8.6%, coronary heart disease (CHD) from 26% to 34%, diabetes from 12% to 16% and cancer from 50% to 59%. In particular, 313/374 and 383/411 participants with hypertension took the medication for elevated blood pressure at each visit, respectively. ACE inhibitors and beta blockers were the two most widely used medications. According to our definition of hypertension, the participants using AHM were considered to be have hypertension. Among the 546 participants, after the first visit, 13 stopped using any AHM and 83 started to use.

**Table 1:** Characteristics of participants from the Normative Aging Study (NAS), 1999–2013 (N = 546) ^a^.

**Characteristics**	**First visit**		**Second visit**
**Age (years)**	71.6 (6.5)		75.4 (6.5)
**DNA methylation age (Horvath, years)**	72.6 (6.7)		74.9 (7.1)
**Age acceleration (Horvath, years)**	0.15 (5.3)		-0.05 (5.6)
**Fasting glucose (mg/dL)**	108.3 (29.2)		105.9 (21.6)
**Total cholesterol (mg/dL)**	198.8 (36.9)		180.4 (37.2)
**Serum triglyceride (mg/dL)**	139.5 (85.3)		125.8 (67.8)
**HDL (mg/dL)**	49.6 (13.4)		48.6 (13.1)
**SBP (mm Hg)**	131.5 (17.3)		124.6 (17.2)
**Smoking status**			
Current smoker	23 (4.2%)		23 (4.2%)
Former smoker	348 (63.7%)		349 (63.9%)
Never smoker	175 (32.1%)		174 (31.9%)
**Body mass index**			
Underweight or normal weight (<25.0)	103 (18.9%)		130 (23.8%)
Overweight (≥25 to <30)	299 (54.7%)		279 (51.1%)
Obese (≥30.0)	144 (26.4%)		137 (25.1%)
**Alcohol consumption ^b^**			
Abstainer	114 (22.5%)		115 (25.1%)
Low (0 to <40 g/d)	355 (70.2%)		319 (69.5%)
Intermediate (40 to <60 g/d)	25 (4.9%)		18 (3.9%)
High (≥60 g/d)	12 (2.4%)		7 (1.5%)
**Physical activity (MET-hours/week) ^c^**			
Low (≤12 kcal/kg hours/week)	321 (61.4%)		307 (64.2%)
Median (12–30 kcal/kg hours/week)	128 (24.5%)		110 (23.0%)
High (≥30 kcal/kg hours/week)	74 (14.1%)		61 (12.8%)
**Major diseases**			
Hypertension	374 (68.5%)		411 (75.3%)
Stroke	32 (5.9%)		47 (8.6%)
Coronary heart disease (CHD)	143 (26.2%)		184 (33.7%)
Diabetes	67 (12.3%)		89 (16.3%)
Cancer	271 (49.6%)		323 (59.2%)
**Use of any antihypertensive medication**			
Any	313 (57.3%)		383 (70.1%)
Calcium channel blockers	66 (12.1%)		84 (15.4%)
ACE inhibitors	143 (26.2%)		204 (37.4%)
Angiotensin receptor antagonists	21 (3.8%)		45 (8.2%)
Alpha blockers	64 (11.7%)		90 (16.5%)
Beta blockers	185 (33.9%)		222 (40.7%)
Diuretics	98 (17.9%)		141 (25.8%)
	**Both visits**		
**Years of education ^d^**			
≤12 years	233 (44.5%)		
13 – 16 years	223 (42.6%)		
>16 years	68 (12.9%)		
**Time between 1st and 2nd visits (years)**	3.86 (1.6)		
**Change rate of age acceleration between visits (year/chronological year)**	-0.03 (1.3)		

a: Mean values (standard deviation) for continuous variables and n (%) for categorical variables;

b: Data missing for 40 and 87 participants at 1^st^ and 2^nd^ visits, respectively;

c: Data missing for 23 and 68 participants at 1^st^ and 2^nd^ visits, respectively;

d: Data missing for 22 participants.

**Figure 1:**
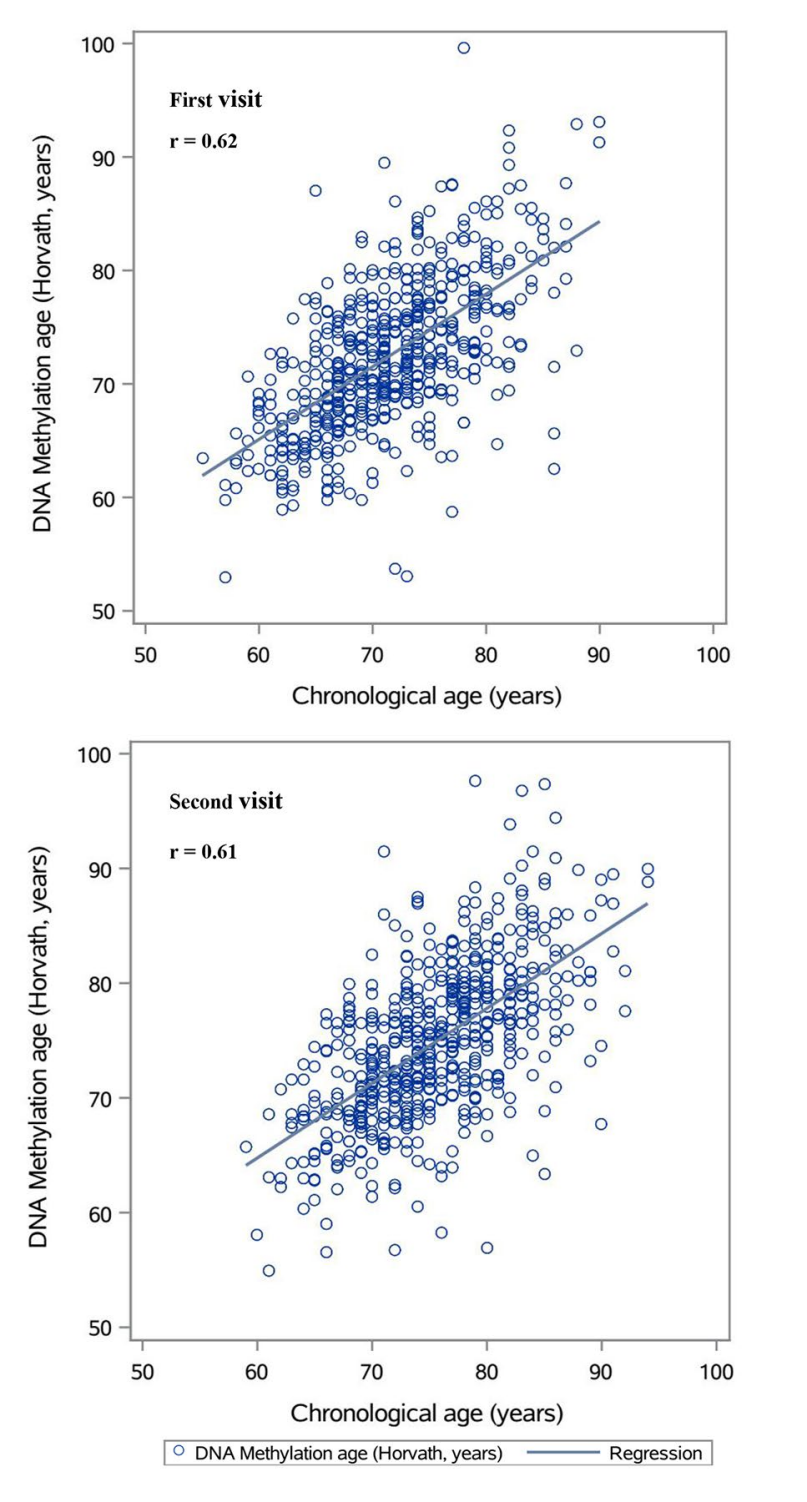
Plots of predicted DNA methylation ages against chronological age.

**Figure 2:**
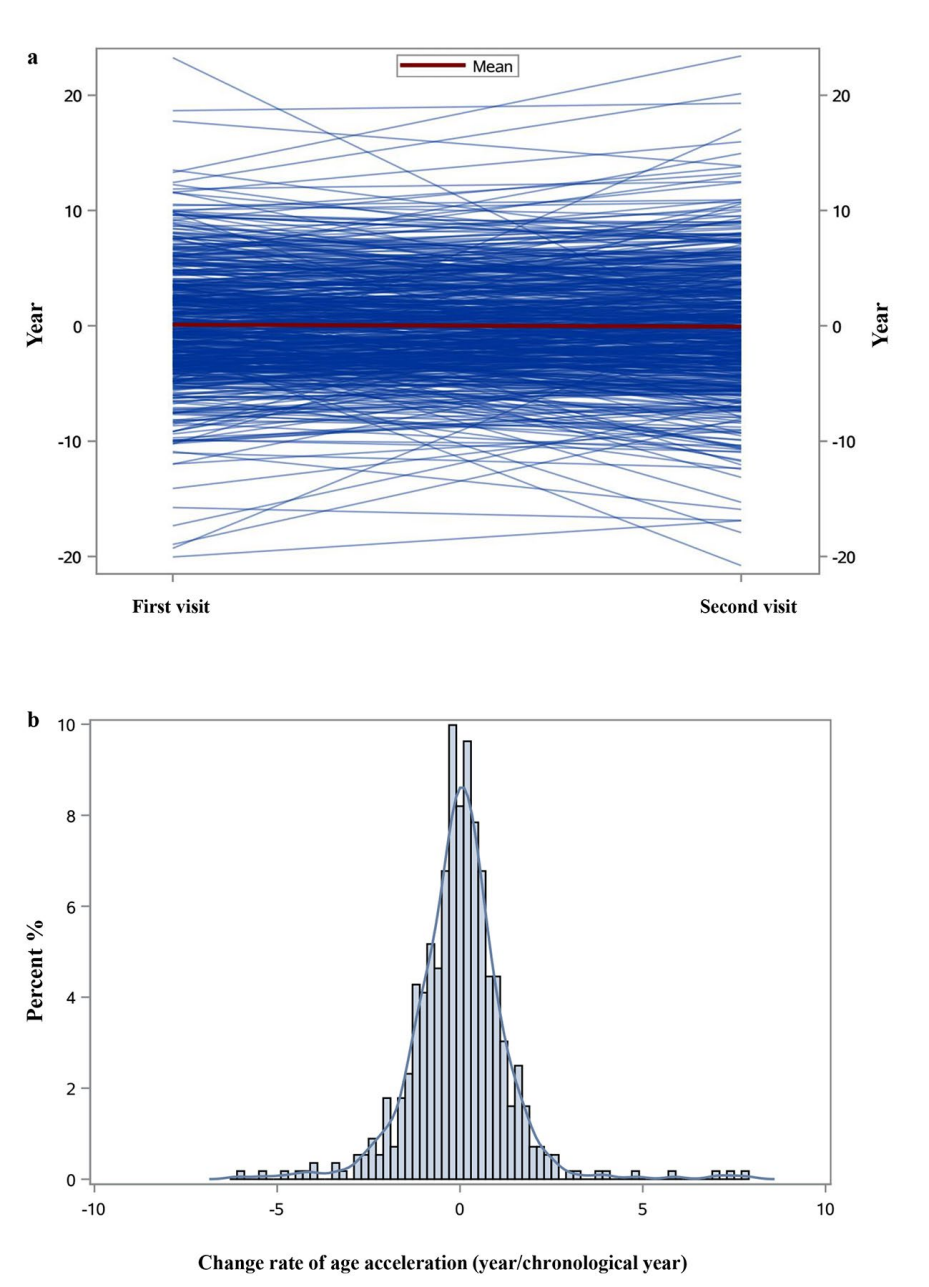
Individual trajectories of age acceleration (**a**) and the distribution of change rate of age acceleration (**b**).

Our main analyses based on the DNAmAge estimated by Horvath’s algorithm. Figure 1 shows that the estimates were highly correlated with chronological age at each visit (Spearman coefficients >0.6). While DNAmAge increased between the first and second visit, the overall AA of the second visit was lower than that of the first visit (Table 1), showing declining trajectories with a crude average Δ_AA_ of about -0.03 year/chronological year. The trajectories of AA and distributions of Δ_AA_ were illustrated in Figure 2. Figure S1 showed strong correlations between the AAs at both visits among those with or without hypertension at both visits (Spearman coefficients ~0.7), while for those who had hypertension or hypertension was controlled after the first visit, their correlation was slightly attenuated (Spearman coefficients = 0.53).

### Associations of hypertension with age acceleration and the change rate of age acceleration

We evaluated the relationship between hypertension and AA at each visit. Participants with hypertension had higher AA (years) than those without (first visit: 0.18 vs. 0.09; second visit: -0.01 vs. -0.18; p-value=0.016). The similar pattern was also observed for Δ_AA_ that participants with hypertension had a higher Δ_AA_ (year/chronological year) compared to those without (first visit: 0.03 vs. -0.14; second visit: 0.01 vs. -0.12; p-value=0.007). We further adjusted for other potential covariates at each visit in a mixed linear model to validate the observed patterns, including age, body max index (BMI), smoking status, alcohol consumption, years of education, physical activity, leukocyte distribution and the batch of microarray experiments in DNA methylation measurement. As shown in Table S1, the correlations of hypertension with AA and Δ_AA_ remained positive, albeit not statistically significant. We also tested the associations of diabetes, metabolic syndrome and commonly measured clinical biomarkers with AA and Δ_AA_ (Table S1 and S2). Despite the statistically significant association between diabetes and Δ_AA_, neither metabolic syndrome nor any biomarkers were strongly associated with AA or Δ_AA_.

### Cross-sectional associations of antihypertensive medication with age acceleration and the change rate of age acceleration

We first investigated the cross-sectional associations of AHM use with AA and Δ_AA_ at each visit. Overall, taking any AHM was significantly associated with higher AA and Δ_AA_ (Table 2). After adjusting for potential covariates at each visit [age, BMI, smoking status, alcohol consumption, years of education, physical activity, leukocyte distribution, total cholesterol, high-density lipoprotein (HDL), triglycerides, fasting glucose, systolic blood pressure (SBP), hypertension, stroke, CHD, diabetes, cancer and the batch of microarray experiments in DNA methylation measurement], people who took any AHM at each visit were about 2.3 years older than the controls in terms of AA, and their AA increased significantly during follow-up at a rate of more than 0.4 year/chronological year compared to the people who did not take any AHMs. However, the effects of specific classes of medications varied. While taking diuretics was negatively associated with AA and Δ_AA_, calcium channel blockers and alpha and beta blockers were positively correlated with AA and Δ_AA_. Angiotensin-converting enzyme inhibitors (ACE inhibitors) and angiotensin receptor antagonists (ARBs) both had negative associations with Δ_AA_, but their relationships with AA were in different directions. An additional subgroup analysis was carried out among people with hypertension at each visit (Table 3), and the use of any AHM remained robustly associated with increased AA and Δ_AA_.

**Table 2:** Cross-sectional associations of the antihypertensive medication use with age acceleration and change rate of age acceleration at first and second visits.

**Visit**	**Medication**	**Medication use**	**N**	**Age acceleration**						**Change rate of age acceleration**				
				**Model 1 ^a^**			**Model 2 ^b^**			**Model 1**			**Model 2**	
				Coefficients(SE)	p-value		Coefficients(SE)	p-value		Coefficients(SE)	p-value		Coefficients(SE)	p-value
**First visit**	Calcium channel blockers	No	480	Ref			Ref			Ref			Ref	
		Yes	66	0.059 (0.723)	0.935		0.381 (0.784)	0.627		0.222(0. 163)	0.175		0.263 (0.184)	0.154
	ACE inhibitors	No	403	Ref			Ref			Ref			Ref	
		Yes	143	0.241 (0.525)	0.646		0.281 (0.605)	0.643		-0.093 (0.119)	0.432		-0.133 (0.142)	0.350
	ARBs	No	525	Ref			Ref			Ref			Ref	
		Yes	21	-0.144 (1.169)	0.902		-0.792 (1.184)	0.504		-0.047 (0.265)	0.858		-0.042 (0.279)	0.881
	Alpha blockers	No	482	Ref			Ref			Ref			Ref	
		Yes	64	1.478 (0.721)	0.041		1.974 (0.769)	0.011		0.055 (0.164)	0.737		0.041 (0.184)	0.821
	Beta blockers	No	361	Ref			Ref			Ref			Ref	
		Yes	185	1.150 (0.499)	0.022		1.291 (0.647)	0.047		0.066 (0.114)	0.562		0.075 (0.153)	0.624
	Diuretics	No	448	Ref			Ref			Ref			Ref	
		Yes	98	-0.068 (0.614)	0.911		-0.100 (0.676)	0.882		-0.095 (0.138)	0.496		-0.001 (0.159)	0.973
	Any antihypertensive medication	No	233	Ref			Ref			Ref			Ref	
		Yes	313	0.935 (0.488)	0.056		2.274 (0.860)	0.009		0.195 (0.111)	0.079		0.433 (0.205)	0.035
**Second**	Calcium channel blockers	No	462	Ref			Ref			Ref			Ref	
**visit**		Yes	84	1.243 (0.681)	0.068		1.035 (0.783)	0.187		0.214 (0.144)	0.137		0.275 (0.163)	0.093
	ACE inhibitors	No	342	Ref			Ref			Ref			Ref	
		Yes	204	-0.293 (0.510)	0.565		-0.340 (0.648)	0.601		-0.045 (0.107)	0.671		-0.036 (0.135)	0.791
	ARBs	No	501	Ref			Ref			Ref			Ref	
		Yes	45	0.790 (0.923)	0.393		0.697 (1.092)	0.524		-0.247 (0.194)	0.203		-0.314 (0.227)	0.167
	Alpha blockers	No	456	Ref			Ref			Ref			Ref	
		Yes	90	0.612 (0.678)	0.367		0.875 (0.814)	0.283		0.034 (0.142)	0.809		0.073 (0.170)	0.668
	Beta blockers	No	324	Ref			Ref			Ref			Ref	
		Yes	222	1.213 (0.506)	0.017		1.406 (0.707)	0.047		0.059 (0.107)	0.581		0.155 (0.148)	0.296
	Diuretics	No	405	Ref			Ref			Ref			Ref	
		Yes	141	-0.136 (0.563)	0.809		-0.047 (0.683)	0.945		-0.029 (0.118)	0.809		-0.050 (0.142)	0.724
	Any antihypertensive medication	No	163	Ref			Ref			Ref			Ref	
		Yes	383	0.659 (0.543)	0.226		2.313 (1.096)	0.035		0.097 (0.114)	0.396		0.467 (0.229)	0.042

a: Model 1: Adjusted for covariates at each visit: age + leukocyte distribution (Houseman algorithm) + random effect (batch effect of DNA methylation measurement). Age acceleration was additionally adjusted for in the model for the change rate of age acceleration;

b: Model 2: Model 1 + BMI + smoking status + alcohol consumption + physical activity + years of education + total cholesterol + HDL + triglycerides + fasting glucose + SBP + hypertension + stroke + CHD + diabetes + cancer.

**Table 3:** Cross-sectional associations of antihypertensive medication use with age acceleration and change of age acceleration at first and second visits among the subpopulation who had hypertension.

**Visit**	**Medication**	**Medication use**	**N**	**Age acceleration^a^**			**Change rate of age acceleration^b^**	
				Coefficients(SE)	p-value		Coefficients(SE)	p-value
**First visit**	Calcium channel blockers	No	308	Ref			Ref	
		Yes	66	-0.390 (0.816)	0.633		0.346 (0.202)	0.089
	ACE inhibitors	No	231	Ref			Ref	
		Yes	143	-0.235 (0.628)	0.709		0.166 (0.156)	0.290
	ARBs	No	353	Ref			Ref	
		Yes	21	-0.144 (1.169)	0.902		0.012 (0.307)	0.968
	Alpha blockers	No	310	Ref			Ref	
		Yes	64	2.087 (0.795)	0.009		-0.048 (0.203)	0.813
	Beta blockers	No	189	Ref			Ref	
		Yes	185	1.410 (0.680)	0.039		0.107 (0.171)	0.531
	Diuretics	No	276	Ref			Ref	
		Yes	98	-0.010 (0.710)	0.989		0.020 (0.177)	0.909
	Any antihypertensive medication	No	61	Ref			Ref	
		Yes	313	2.378 (0.898)	0.009		0.497 (0.228)	0.030
**Second visit**	Calcium channel blockers	No	327	Ref			Ref	
		Yes	84	0.942 (0.794)	0.236		0.307 (0.153)	0.046
	ACE inhibitors	No	207	Ref			Ref	
		Yes	204	-0.215 (0.657)	0.743		0.016 (0.128)	0.899
	ARBs	No	366	Ref			Ref	
		Yes	45	0.687 (1.084)	0.527		-0.285 (0.215)	0.187
	Alpha blockers	No	329	Ref			Ref	
		Yes	82	0.584 (0.867)	0.501		0.032 (0.171)	0.851
	Beta blockers	No	189	Ref			Ref	
		Yes	222	1.287 (0.715)	0.073		0.123 (0.142)	0.388
	Diuretics	No	270	Ref			Ref	
		Yes	141	0.010 (0.685)	0.989		-0.031 (0.136)	0.818
	Any antihypertensive medication	No	36	Ref			Ref	
		Yes	375	2.313 (1.214)	0.058		0.430 (0.235)	0.069

a: Adjusted for covariates at each visit: age + leucocyte distribution (Houseman algorithm) + BMI + smoking status + alcohol consumption + physical activity + years of education + total cholesterol + HDL + triglycerides + fasting glucose + SBP + stroke + CHD + diabetes + cancer + random effect (batch effect of methylation measurement);

b: Additionally adjusted for age acceleration at each visit.

Table S3a showed sensitivity analyses for the cross-sectional associations of any AHM use with the AA and Δ_AA_ of Hannum DNAmAge and DNA methylation Phenotypic age (DNAmPhenoAge). Taking any AHMs showed positive correlations with the AA of each biomarker and significantly associated with both forms of AA at the first visit. People who took medications were about 2.2 years/1.7 years older in people compared to the controls, respectively. Even though taking any AHMs was not robustly associated with the Δ_AA_ of neither biomarker, the effects were in the same direction as we identified for the Horvath DNAmAge.

### Longitudinal associations of antihypertensive medication with age acceleration and the change rate of age acceleration

We further examined the longitudinal association between AHM use and Δ_AA_ (Table 4). People that with continuous medication use were older than that never used (76.6 vs. 73.7 years). After controlling for the potential covariates at the first visit, taking AHM showed positive correlations with the AA and Δ_AA_. Compared to people who never took any AHM, individuals who started to take AHM after the first visit had an increased Δ_AA_ of about 0.3 year/chronological year, and individuals with continuous AHM use had an increased Δ_AA_ of about 0.6 year/chronological year. Consistent with this finding, stopping taking ACE inhibitors, ARBs, and alpha and beta blockers showed negative correlations with Δ_AA_, compared to continuous use. A subgroup analysis among 313 participants with hypertension at first visit yielded a similar pattern (Table 5). After fully controlling for potential covariates, stopped taking AHM was correlated with a declining Δ_AA_ compared to the continued AHM use after the first visit albeit not statistically significant.

**Table 4:** Associations between antihypertensive medication use and change rate of age acceleration from first to second visit.

**Medication**	**Change of medication use**	**N**	**Change rate of age acceleration**				
			**Model 1 ^a^**			**Model 2^ b^**	
			Coefficients(SE)	p-value		Coefficients(SE)	p-value
Calcium channel blockers	Never used	440	Ref			Ref	
	Stopped use after first visit	22	0.103 (0.272)	0.706		0.264 (0.304)	0.386
	Started use after first visit	40	0.437 (0.201)	0.030		0.405 (0.216)	0.062
	Continuous use	44	0.338 (0.194)	0.083		0.338 (0.214)	0.116
ACE inhibitors	Never used	327	Ref			Ref	
	Stopped use after first visit	15	-0.121 (0.316)	0.701		-0.026 (0.348)	0.942
	Started use after first visit	76	-0.081 (0.159)	0.614		0.033 (0.185)	0.857
	Continuous use	128	0.103 (0.128)	0.419		0.163 (0.156)	0.297
ARBs	Never used	496	Ref			Ref	
	Stopped use after first visit	5	-0.223 (0.535)	0.677		-0.250 (0.556)	0.654
	Started use after first visit	29	-0.216 (0.235)	0.359		-0.145 (0.276)	0.599
	Continuous use	16	-0.006 (0.303)	0.983		0.011 (0.319)	0.972
Alpha blockers	Never used	442	Ref			Ref	
	Stopped use after first visit	14	-0.422( 0.325)	0.195		-0.330 (0.371)	0.375
	Started use after first visit	40	0.105 (0.202)	0.605		0.269 (0.222)	0.226
	Continuous use	50	0.065 (0.185)	0.727		0.063 (0.201)	0.753
Beta blockers	Never used	300	Ref			Ref	
	Stopped use after first visit	24	-0.586 (0.268)	0.029		-0.598 (0.300)	0.047
	Started use after first visit	61	0.180 (0.169)	0.288		0.290 (0.185)	0.117
	Continuous use	161	0.193 (0.122)	0.114		0.471 (0.164)	0.100
Diuretics	Never used	385	Ref			Ref	
	Stopped use after first visit	20	0.183 (0.277)	0.509		0.010 (0.293)	0.972
	Started use after first visit	63	-0.017 (0.169)	0.921		-0.019 (0.186)	0.918
	Continuous use	78	0.068 (0.154)	0.658		-0.016 (0.181)	0.932
Any antihypertensive medication	Never used	150	Ref			Ref	
	Stopped use after first visit	13	-0.117 (0.374)	0.754		0.006 (0.433)	0.989
	Started use after first visit	83	0.135 (0.168)	0.422		0.285 (0.185)	0.124
	Continuous use	300	0.258 (0.127)	0.043		0.596 (0.220)	0.008

a: Model 1: Adjusted for: age (first visit) + age acceleration (first visit) + leukocyte distribution (first visit, Houseman algorithm) + random effect (batch effect of methylation measurement at first visit);

b: Model 2: Model 1 + BMI (first visit) + smoking status (first visit) + alcohol consumption (first visit) + physical activity (first visit) + years of education (first visit) + total cholesterol (first visit) + HDL (first visit) + triglycerides (first visit) + fasting glucose (first visit) + SBP (first visit) + hypertension (first visit) + stroke (first visit) + CHD (first visit) + diabetes (first visit) + cancer (first visit).

**Table 5:** Associations between stopping antihypertensive medication use and change rate of age acceleration among the subpopulation that used the antihypertensive medications at first visit.

**Medication**	**Change of medication use**	**N**	**Changes of age acceleration**				
			**Model 1 ^a^**			**Model 2 ^b^**	
			Coefficients(SE)	p-value		Coefficients(SE)	p-value
Calcium channel blockers	Stopped use after first visit	22	-0.415 (0.316)	0.196		-0.143 (0.442)	0.751
	Continuous use	44	Ref			Ref	
ACE inhibitors	Stopped use after first visit	15	-0.308 (0.322)	0.342		-0.212 (0.369)	0.567
	Continuous use	128	Ref			Ref	
ARBs	Stopped use after first visit	5	-1.724 (0.831)	0.077		-0.050 (3.331)	0.985
	Continuous use	16	Ref			Ref	
Alpha blockers	Stopped use after first visit	14	-0.482 (0.294)	0.109		-0.040 (0.569)	0.945
	Continuous use	50	Ref			Ref	
Beta blockers	Stopped use after first visit	24	-0.731 (0.333)	0.030		-0.824 (0.401)	0.042
	Continuous use	161	Ref			Ref	
Diuretics	Stopped use after first visit	20	0.156 (0.335)	0.642		-0.263 (0.407)	0.521
	Continuous use	78	Ref			Ref	
Any antihypertensive medication	Stopped use after first visit	13	-0.317 (0.391)	0.418		-0.585 (0.434)	0.180
	Continuous use	300	Ref			Ref	

a: Model 1: Adjusted for age (first visit) + age acceleration (first visit) + leukocyte distribution (first visit, Houseman algorithm) + random effect (batch effect of methylation measurement at first visit);

b: Model 2: Model 1 + BMI (first visit) + smoking status (first visit) + alcohol consumption (first visit) + physical activity (first visit) + years of education (first visit) + total cholesterol (first visit) + HDL (first visit) + triglycerides (first visit) + fasting glucose (first visit) + SBP (first visit) + hypertension (first visit) + stroke (first visit) + CHD (first visit) + diabetes (first visit) + cancer (first visit).

Same longitudinal tests were also performed for the Δ_AA_ of Hannum DNAmAge and DNAmPhenoAge as sensitivity analyses in Table S3b. The Δ_AA_ of neither biomarker showed significant associations with the change of AHM use, but an increasing pattern was still observed for the people with continuous AHMs use in comparison to the people who never used the AHM for each biomarker.

## DISCUSSION

In the present study, we investigated the cross-sectional and longitudinal associations of AHM use with the AA of Horvath DNAmAge in a longitudinal study of older male participants examined over two visits. Even though a general decrease of AA was observed between the two visits, after fully adjusting for hypertension and other potential covariates, any AHM use showed a cross-sectional significant association with higher AA at each visit, as well as a longitudinal association with increased Δ_AA_ between visits. Particularly, relative to participants who never took any AHM, individuals with continuous AHM use had a higher Δ_AA_ of 0.6 year/chronological year. Additional sensitivity analyses on another two DNA methylation-based biomarkers (Hannum DNAmAge & DNAmPhenoAge) showed the similar patterns with the use of AHM as the Horvath DNAmAge.

Recently, Marioni et al. found that DNAmAge increases at a slower rate than chronological age across the life course in five independent cohorts, especially in the older population [21], which suggested that, overall, AA declines as people get older. The global decreasing pattern of AA across the two visits observed in our study is in line with this finding. This pattern may also be explained by survival bias, due to the higher probability that healthier participants with relatively lower AA may stay longer in longitudinal studies. After adjusting for hypertension and other potential covariates that might affect the DNAmAge, we surprisingly found that taking AHMs was associated with faster AA. This finding is at variance with the declining pattern of DNAmAge across the lifespan and is not consistent with the preventive effects of AHMs against age-related health outcomes caused by hypertension [18,19]. Several explanations may account for this discrepancy.

First, measurement bias or inaccuracies may affect the comparability of longitudinal AA and Δ_AA_ estimates. Nevertheless, we have restricted this technical bias to the greatest extent by adjusting for the batch of DNA methylation measurement as a random effect and normalizing the methylation profiles with Horvath’s internal normalization method. Furthermore, selection bias due to differential survival rate may cause an underrepresentation of participants with higher AA [22], and may lead to underestimation or even contradicting results of the effect of AHM use on AA and Δ_AA_, and distort their associations towards the opposite. In our analyses, we accounted for potential selection bias due to the loss of follow-up using inverse probability weighting (IPW) [23]. Point estimates were similar between models using and not using IPW, indicating that little selection bias was introduced due to the loss to follow-up. Additionally, since the use of AHMs is usually treated as the indicator of hypertension severity, our study might report a proxy outcome that indeed reflected the impact of hypertension on aging (confounding by indication). Due to this concern, despite the weak positive but not significant pattern we observed between hypertension and the AA (Δ_AA_), we additionally adjusted for SBP and hypertension in the fully-adjusted analysis model and performed another sensitivity analysis by adding the interaction term of hypertension and AHMs (data not shown). Neither of the two adjustments altered the patterns of taking AHMs with AA and Δ_AA_ in any relevant manner. In the meanwhile, people might stop taking AHMs after reducing the blood pressure through changing lifestyles and exercises, two factors that are likely to decrease the Δ_AA_. After controlling for the factors, we still observed a declining pattern between stopping taking any AHM and Δ_AA_, albeit not significant due to the limited sample size.

One biologically plausible explanation for this inconsistent observation is a potential causal connection among aging, epigenetic biomarkers of age, hypertension and AHM use. We speculate that in these four-corner relationships, aging independently leads to the change of epigenetic biomarkers of age (e.g., DNAmAge, DNAmPhenoAge) and hypertension via two separate pathways. Aging could cause the change of epigenetic biomarkers of age by altering the methylation levels of age-related CpG sites. In parallel, aging could prompt hypertension via a “vicious cycle”, which consists of inflammation, oxidative stress and endothelial dysfunction, and might not be closely related to age-related DNA methylation changes [16,17]. Taking AHMs could control blood pressure and reduce the risks of aging-related diseases, such as CVD, kidney failure and dementia, which are caused by hypertension. Nevertheless, as the causation between aging and hypertension is one-way, reducing blood pressure using AHM might not reversely affect biological aging by bringing the aberrant changes of age-related CpG sites back to normal levels. On the contrary, the AHMs’ potential side effects, such as abnormal glucose and lipid metabolism [24] and psychological/cognitive disorders [25,26], might have the potential to accelerate epigenetic biomarkers of age as suggested by previous studies [6,10,27]. However, this hypothesis needs to be evaluated by further research with larger populations and multiple follow-ups along with corresponding causal inferences and functional tests.

It is worth noting that the effects of specific medications on AA and Δ_AA_ were not all unfavorable. In our study, ACE inhibitors, ARBs, and diuretics showed weak negative correlation with AA and Δ_AA_, while beta blockers showed a strong aging accelerating effect. Beyond the effects from specific medications, we should not ignore the adverse drug reactions (ADRs) from combined and inappropriate medication use [28], given most of the participants with hypertension took multiple medications at the same time. ADRs could become more severe and frequent in the elderly due to age-dependent pharmacodynamics and pharmacokinetic changes promoting drug-drug or drug-disease interactions, and such reactions could directly (or indirectly) facilitate the development of aging-related health outcomes including frailty and all-cause mortality [29,30]. This effect might play another key role in accelerating DNAmAge if the ADRs are not identified and treated with further changes in prescriptions.

Major strengths of the present study include the relatively large sample size and repeated measurements with detailed information on a broad range of covariates in a large cohort study. We also acknowledge several limitations in the interpretation of results. First, shifts of leukocyte distribution might affect the DNA methylation changes in whole blood samples [31]. Hence, we adjusted for leukocyte distribution by the Houseman algorithm to restrict potential confounding from differential blood counts to the greatest possible extent [32]. Nevertheless, residual confounding by this and other factors cannot be excluded entirely for the longitudinal analysis. Furthermore, the selected study participants were Caucasians and all male, which limits the generalizability of our results to other racial/ethnic groups and women. Finally, although our overall sample size was relatively large, some of the nonsignificant results may have been due to the lack of statistical power with the relatively smaller size of subsamples for specific AHMs. And given those subgroup analyses did not appear as robust as the whole population and showed contradicted patterns, there might be a risk of getting false positive findings, further research with a bigger sample size and longer multiple follow-ups are warranted to validate our results by eliminating the possibility of false positives.

In summary, we observed cross-sectional and longitudinal associations of any AHM use with increased AA and Δ_AA_. Our findings suggest that controlling blood pressure by taking medications might not be able to reduce the accelerated epigenetic aging, on the contrary, was associated with accelerated DNAmAge. This study partly reveals the relationship between AHMs and biological aging, and also underlines that DNAmAge and AA may not be able to capture the preventive effects of AHMs that reduce cardiovascular risks and mortality. Future investigations are required to confirm our findings and to elucidate the causal relationship between AHM use and DNAmAge, as well as the underlying pathophysiological mechanisms.

## MATERIALS AND METHODS

### Study design and population

The NAS study is an ongoing longitudinal study of aging, established by the U.S. Department of Veterans Affairs in 1963. Details of this study have been published previously [33]. Briefly, the NAS is a closed cohort of 2,280 male veterans from the Greater Boston area. They were enrolled after an initial health screening that determined that they were free of known chronic medical conditions. Blood samples were collected from 657 participants, most of whom were visited up to 4 times between 1999 and 2013. Participants have been reevaluated every 3–5 years on a continuous rolling basis using detailed on-site physical examinations and questionnaires. We restricted the current analysis to the data of the first two visits of 546 Caucasian participants (aged 55-85 years) who had been visited twice at least, in order to control for the heterogeneity of race and to analyze the change of AA longitudinally. The NAS study was approved by the Department of Veterans Affairs Boston Healthcare System, and written informed consent was obtained from each subject prior to participation.

### Data collection

As previously described [34], at each visit, participants were asked to provide detailed information about their lifestyles, dietary habits, activity levels, and demographic factors. Height and weight were measured and were used to calculate body mass index (BMI, in kg/m^2^). Blood samples were collected for assessing blood-based biomarkers. Participants’ status of major diseases was assessed based on the medical history and physicians’ diagnosis. In particular, hypertension was defined as a measured SBP of ≥140 mmHg, a measured diastolic blood pressure (DBP) of ≥90 mm Hg, or participants’ use of AHMs [35]. SBP and DBP were measured by a physician, and the AHMs included calcium channel blockers, ACE inhibitors, ARBs, alpha blockers, beta blockers and diuretics.

### DNA methylation data

DNA of whole blood samples were collected between 1999 and 2013. As previously described [36,37], we used the QIAamp DNA Blood Kit (Qiagen, CA, USA) to extract DNA from buffy coat, and performed bisulfite conversion with the EZ-96 DNA Methylation Kit (Zymo Research, CA, USA). To minimize batch effects, we randomized chips across plates and randomized samples based on a 2-stage age-stratified algorithm so that age distributed similarly across chips and plates. We measured DNA methylation of CpG probes using Illumina HumanMethylation450 BeadChip for all the samples from both visits. After quality control, the remaining samples were preprocessed using the Illumina-type background correction, dye-bias adjustment and BMIQ normalization [38], which were used to generate methylation status. The methylation status of a specific CpG site was quantified as a β value ranging from 0 (no methylation) to 1 (full methylation).

A total of 353 CpG sites were retrieved from the methylation profiles for the estimation of DNAmAge for each participant based on the algorithm proposed by Horvath [3]. This algorithm was derived from a range of tissues and cell types using 353 probes targeted in the Illumina 27k and 450k methylation arrays. In this study, we performed the estimation by using the online calculator, where background-corrected beta values were pre-processed by the calculator’s internal normalization method [3]. AA was determined as discrepancies between methylation and chronological age in the form of residuals, which have a mean of 0 and represent positive and negative deviations from chronological age in years. The residuals were calculated by a linear regression procedure in which DNA methylation age was the outcome and chronological age was the independent variable. The Δ_AA_ for each participant between the two visits was determined as:

$$\Delta_{\text{AA}}=\frac{\text{AA of second visit (year) - AA of first visit (year)}}{\text{Time between first and second visit (year)}}$$ (1)

with a unit of year/chronological year.

### Statistical analysis

Study population of each visit was described with respect to major socio-demographic characteristics, lifestyle factors and detailed AHM use. The correlation between estimated DNAmAge and chronological age at each visit was evaluated by Spearman correlation coefficient.

We first tested the cross-sectional association of hypertension with AA at each visit and the Δ_AA_ between the two visits. A linear mixed model with AA (Δ_AA_) as outcome was employed, controlling for covariates that have been reported to be associated with DNA methylation changes or with the use of AHM, including age (years), BMI [kg/m^2^, underweight (<18.5, < 1% of the study population) or normal weight (18.5 to <25), overweight (25 to <30), obese (≥30)], alcohol consumption [abstainer, low (0 to <40 g/d), intermediate (40 to <60 g/d), high (≥60 g/d)], smoking (current/ former/ never smoker), education (≤12 years, 13 – 16 years, >16 years), physical activity [metabolic equivalent of task (MET), low (≤12 kcal/kg hours/week), median (12–30 kcal/kg hours/week), high (≥30 kcal/kg hours/week)] and leukocyte distribution (Houseman algorithm [32]). The batch of microarray experiments in DNA methylation measurement was controlled for as the random effect. Corresponding AA was additionally adjusted for in the analyses of Δ_AA_ at each visit.

We then investigated the cross-sectional associations of AHM use with AA and Δ_AA_ at each visit in two mixed linear regression models. Model 1 adjusted for age, leukocyte distribution and the random batch effect, and Model 2 further adjusted for BMI, smoking status, alcohol consumption, physical activity, education, total cholesterol (mg/dL), HDL (mg/dL), triglycerides (mg/dL), fasting glucose (mg/dL), SBP (mm Hg), hypertension, stroke, coronary heart disease, diabetes and cancer (yes/no). AA was additionally adjusted for in the analyses of Δ_AA_ at each visit.

Finally, we examined the longitudinal association between AHM use and the Δ_AA_. AHM use in the longitudinal analysis was classified as: never used, stopped use after first visit, started use after first visit and continuous use according to the reports of AHM use between the two visits, and was treated as a predictor in the analysis models. Two mixed linear regression models were used, adjusting for the same covariates described above.

### Sensitivity analyses

We further performed sensitivity analyses for the main results by using another two DNA methylation based aging biomarkers: DNAmAge estimated by Hanumm et al.’s algorithm [39], and DNAmPhenoAge recently developed by Morgan et al. as an “update” of the Horvath DNAmAge for the lifespan [40].

Hanumm DNAmAge was estimated based on 71 age-related CpG sites reported in 2013 [39] and determined as the sum of the methylation beta values multiplied by the reported effect sizes of the predictors. AA of Hannum’s DNAmAge was also determined as discrepancies between DNA methylation and chronological age in the form of residuals.

Another batch of 513 CpG sites was retrieved for the estimation of DNAmPhenoAge for each participant based on the algorithm proposed by Levine et al. [40]. With the coefficient and intercept values provided by the authors, we estimated the DNAmPhenoAge as:

DNAmPhenoAge = 𝐶𝑝𝐺_1_ × 𝛽_1_ + 𝐶𝑝𝐺_2_ × 𝛽_2_ +⋯ 𝐶𝑝𝐺_513_ × 𝛽_513_ + intercept.

As defined by the authors [40], difference between Phenotypic and chronological age (DNAmPhenoAge – chronological age) was defined as the AA of DNAmPhenoAge. The Δ_AA_ for the two aging biomarkers between the two visits was also determined by the formula (1).

We tested the cross-sectional and longitudinal associations of the AA and Δ_AA_ of the two biomarkers with the use of any AHM using the same analysis models employed in the main analyses for Horvath DNAmAge.

Data cleaning and all aforementioned analyses were performed by SAS version 9.4 (SAS Institute Inc., Cary, NC, USA), and all statistical tests were two-sided with p-values of <0.05.

## SUPPLEMENTARY MATERIAL

Supplementary Table S1

Supplementary Table S2

Supplementary Table S3

Supplementary Figure S1

## References

[1] Jones MJ, Goodman SJ, Kobor MS. DNA methylation and healthy human aging. Aging Cell. 2015; 14:924–32. 10.1111/acel.12349 25913071 PMC4693469

[2] Jung M, Pfeifer GP. Aging and DNA methylation. BMC Biol. 2015; 13:7. 10.1186/s12915-015-0118-4 25637097 PMC4311512

[3] Horvath S. DNA methylation age of human tissues and cell types. Genome Biol. 2013; 14:R115. 10.1186/gb-2013-14-10-r115 24138928 PMC4015143

[4] Horvath S, Erhart W, Brosch M, Ammerpohl O, von Schönfels W, Ahrens M, Heits N, Bell JT, Tsai PC, Spector TD, Deloukas P, Siebert R, Sipos B, et al. Obesity accelerates epigenetic aging of human liver. Proc Natl Acad Sci USA. 2014; 111:15538–43. 10.1073/pnas.1412759111 25313081 PMC4217403

[5] Boks MP, van Mierlo HC, Rutten BP, Radstake TR, De Witte L, Geuze E, Horvath S, Schalkwyk LC, Vinkers CH, Broen JC, Vermetten E. Longitudinal changes of telomere length and epigenetic age related to traumatic stress and post-traumatic stress disorder. Psychoneuroendocrinology. 2015; 51:506–12. 10.1016/j.psyneuen.2014.07.011 25129579

[6] Marioni RE, Shah S, McRae AF, Ritchie SJ, Muniz-Terrera G, Harris SE, Gibson J, Redmond P, Cox SR, Pattie A, Corley J, Taylor A, Murphy L, et al. The epigenetic clock is correlated with physical and cognitive fitness in the Lothian Birth Cohort 1936. Int J Epidemiol. 2015; 44:1388–96. 10.1093/ije/dyu277 25617346 PMC4588858

[7] Horvath S, Gurven M, Levine ME, Trumble BC, Kaplan H, Allayee H, Ritz BR, Chen B, Lu AT, Rickabaugh TM, Jamieson BD, Sun D, Li S, et al. An epigenetic clock analysis of race/ethnicity, sex, and coronary heart disease. Genome Biol. 2016; 17:171. 10.1186/s13059-016-1030-0 27511193 PMC4980791

[8] Perna L, Zhang Y, Mons U, Holleczek B, Saum KU, Brenner H. Epigenetic age acceleration predicts cancer, cardiovascular, and all-cause mortality in a German case cohort. Clin Epigenetics. 2016; 8:64. 10.1186/s13148-016-0228-z 27274774 PMC4891876

[9] Gao X, Zhang Y, Brenner H. Associations of Helicobacter pylori infection and chronic atrophic gastritis with accelerated epigenetic ageing in older adults. Br J Cancer. 2017; 117:1211–14. 10.1038/bjc.2017.314 28898235 PMC5674108

[10] Quach A, Levine ME, Tanaka T, Lu AT, Chen BH, Ferrucci L, Ritz B, Bandinelli S, Neuhouser ML, Beasley JM, Snetselaar L, Wallace RB, Tsao PS, et al. Epigenetic clock analysis of diet, exercise, education, and lifestyle factors. Aging (Albany NY). 2017; 9:419–46. 10.18632/aging.101168 28198702 PMC5361673

[11] Fiorito G, Polidoro S, Dugué PA, Kivimaki M, Ponzi E, Matullo G, Guarrera S, Assumma MB, Georgiadis P, Kyrtopoulos SA, Krogh V, Palli D, Panico S, et al. Social adversity and epigenetic aging: a multi-cohort study on socioeconomic differences in peripheral blood DNA methylation. Sci Rep. 2017; 7:16266. 10.1038/s41598-017-16391-5 29176660 PMC5701128

[12] Dugue PA, Bassett JK, Joo JE, Baglietto L, Jung CH, Ming Wong E, Fiorito G, Schmidt D, Makalic E, Li S, Moreno-Betancur M, Buchanan DD, Vineis P, et al. Association of DNA Methylation-Based Biological Age with Health Risk Factors, and Overall and Cause-Specific Mortality. Am J Epidemiol. 2018; 187:529–38. 10.1093/aje/kwx291 29020168

[13] Gao X, Zhang Y, Breitling LP, Brenner H. Relationship of tobacco smoking and smoking-related DNA methylation with epigenetic age acceleration. Oncotarget. 2016; 7:46878–89. 10.18632/oncotarget.9795 27276709 PMC5216910

[14] Schulz E, Gori T, Münzel T. Oxidative stress and endothelial dysfunction in hypertension. Hypertens Res. 2011; 34:665–73. 10.1038/hr.2011.39 21512515

[15] Schulz E, Jansen T, Wenzel P, Daiber A, Münzel T. Nitric oxide, tetrahydrobiopterin, oxidative stress, and endothelial dysfunction in hypertension. Antioxid Redox Signal. 2008; 10:1115–26. 10.1089/ars.2007.1989 18321209

[16] Sun Z. Aging, arterial stiffness, and hypertension. Hypertension. 2015; 65:252–56. 10.1161/HYPERTENSIONAHA.114.03617 25368028 PMC4288978

[17] Buford TW. Hypertension and aging. Ageing Res Rev. 2016; 26:96–111. 10.1016/j.arr.2016.01.007 26835847 PMC4768730

[18] Neal B, MacMahon S, Chapman N, and Blood Pressure Lowering Treatment Trialists’ Collaboration. Effects of ACE inhibitors, calcium antagonists, and other blood-pressure-lowering drugs: results of prospectively designed overviews of randomised trials. Blood Pressure Lowering Treatment Trialists’ Collaboration. Lancet. 2000; 356:1955–64. 10.1016/S0140-6736(00)03307-9 11130523

[19] Turnbull F, and Blood Pressure Lowering Treatment Trialists’ Collaboration. Effects of different blood-pressure-lowering regimens on major cardiovascular events: results of prospectively-designed overviews of randomised trials. Lancet. 2003; 362:1527–35. 10.1016/S0140-6736(03)14739-3 14615107

[20] Yasar S, Schuchman M, Peters J, Anstey KJ, Carlson MC, Peters R, and Review of Human Studies and Clinical Trials. Relationship Between Antihypertensive Medications and Cognitive Impairment: part I. Curr Hypertens Rep. 2016; 18:67. 10.1007/s11906-016-0674-1 27492370 PMC4975763

[21] Marioni RE, Suderman M, Chen BH, Horvath S, Bandinelli S, Morris T, Beck S, Ferrucci L, Pedersen NL, Relton CL, Deary IJ, Hägg S. Tracking the Epigenetic Clock Across the Human Life Course: A Meta-analysis of Longitudinal Cohort Data. J Gerontol A Biol Sci Med Sci. 2018. 10.1093/gerona/gly060 29718110 PMC6298183

[22] Howe CJ, Cole SR, Lau B, Napravnik S, Eron JJ Jr. Selection Bias Due to Loss to Follow Up in Cohort Studies. Epidemiology. 2016; 27:91–97. 10.1097/EDE.0000000000000409 26484424 PMC5008911

[23] Seaman SR, White IR. Review of inverse probability weighting for dealing with missing data. Stat Methods Med Res. 2013; 22:278–95. 10.1177/0962280210395740 21220355

[24] Lardinois CK, Neuman SL. The effects of antihypertensive agents on serum lipids and lipoproteins. Arch Intern Med. 1988; 148:1280–88. 10.1001/archinte.1988.00380060044012 2897834

[25] Hamer M, Batty GD, Stamatakis E, Kivimaki M. Hypertension awareness and psychological distress. Hypertension. 2010; 56:547–50. 10.1161/HYPERTENSIONAHA.110.153775 20625078 PMC3319302

[26] Brody DS. Psychological distress and hypertension control. J Human Stress. 1980; 6:2–6. 10.1080/0097840X.1980.9935012 7373028

[27] Zannas AS, Arloth J, Carrillo-Roa T, Iurato S, Röh S, Ressler KJ, Nemeroff CB, Smith AK, Bradley B, Heim C, Menke A, Lange JF, Brückl T, et al. Lifetime stress accelerates epigenetic aging in an urban, African American cohort: relevance of glucocorticoid signaling. Genome Biol. 2015; 16:266. 10.1186/s13059-015-0828-5 26673150 PMC4699359

[28] Davies EC, Green CF, Taylor S, Williamson PR, Mottram DR, Pirmohamed M. Adverse drug reactions in hospital in-patients: a prospective analysis of 3695 patient-episodes. PLoS One. 2009; 4:e4439. 10.1371/journal.pone.0004439 19209224 PMC2635959

[29] Saum KU, Schöttker B, Meid AD, Holleczek B, Haefeli WE, Hauer K, Brenner H. Is Polypharmacy Associated with Frailty in Older People? Results From the ESTHER Cohort Study. J Am Geriatr Soc. 2017; 65:e27–32. 10.1111/jgs.14718 28024089

[30] Schöttker B, Saum KU, Muhlack DC, Hoppe LK, Holleczek B, Brenner H. Polypharmacy and mortality: new insights from a large cohort of older adults by detection of effect modification by multi-morbidity and comprehensive correction of confounding by indication. Eur J Clin Pharmacol. 2017; 73:1041–48. 10.1007/s00228-017-2266-7 28540438

[31] Schwartz J, Weiss ST. Cigarette smoking and peripheral blood leukocyte differentials. Ann Epidemiol. 1994; 4:236–42. 10.1016/1047-2797(94)90102-3 8055125

[32] Houseman EA, Accomando WP, Koestler DC, Christensen BC, Marsit CJ, Nelson HH, Wiencke JK, Kelsey KT. DNA methylation arrays as surrogate measures of cell mixture distribution. BMC Bioinformatics. 2012; 13:86. 10.1186/1471-2105-13-86 22568884 PMC3532182

[33] Bell B, Rose CL, Damon A. The Normative Aging Study: an interdisciplinary and longitudinal study of health and aging. Aging Hum Dev. 1972; 3:5–17. 10.2190/GGVP-XLB5-PC3N-EF0G

[34] Mordukhovich I, Coull B, Kloog I, Koutrakis P, Vokonas P, Schwartz J. Exposure to sub-chronic and long-term particulate air pollution and heart rate variability in an elderly cohort: the Normative Aging Study. Environ Health. 2015; 14:87. 10.1186/s12940-015-0074-z 26546332 PMC4636903

[35] Nyhan MM, Coull BA, Blomberg AJ, Vieira CL, Garshick E, Aba A, Vokonas P, Gold DR, Schwartz J, Koutrakis P. Associations Between Ambient Particle Radioactivity and Blood Pressure: The NAS (Normative Aging Study). J Am Heart Assoc. 2018; 7:e008245. 10.1161/JAHA.117.008245 29545261 PMC5907574

[36] Panni T, Mehta AJ, Schwartz JD, Baccarelli AA, Just AC, Wolf K, Wahl S, Cyrys J, Kunze S, Strauch K, Waldenberger M, Peters A. Genome-Wide Analysis of DNA Methylation and Fine Particulate Matter Air Pollution in Three Study Populations: KORA F3, KORA F4, and the Normative Aging Study. Environ Health Perspect. 2016; 124:983–90. 10.1289/ehp.1509966 26731791 PMC4937859

[37] Dai L, Mehta A, Mordukhovich I, Just AC, Shen J, Hou L, Koutrakis P, Sparrow D, Vokonas PS, Baccarelli AA, Schwartz JD. Differential DNA methylation and PM_2.5_ species in a 450K epigenome-wide association study. Epigenetics. 2017; 12:139–48. 10.1080/15592294.2016.1271853 27982729 PMC5330435

[38] Teschendorff AE, Marabita F, Lechner M, Bartlett T, Tegner J, Gomez-Cabrero D, Beck S. A beta-mixture quantile normalization method for correcting probe design bias in Illumina Infinium 450 k DNA methylation data. Bioinformatics. 2013; 29:189–96. 10.1093/bioinformatics/bts680 23175756 PMC3546795

[39] Hannum G, Guinney J, Zhao L, Zhang L, Hughes G, Sadda S, Klotzle B, Bibikova M, Fan JB, Gao Y, Deconde R, Chen M, Rajapakse I, et al. Genome-wide methylation profiles reveal quantitative views of human aging rates. Mol Cell. 2013; 49:359–67. 10.1016/j.molcel.2012.10.016 23177740 PMC3780611

[40] Levine ME, Lu AT, Quach A, Chen BH, Assimes TL, Bandinelli S, Hou L, Baccarelli AA, Stewart JD, Li Y, Whitsel EA, Wilson JG, Reiner AP, et al. An epigenetic biomarker of aging for lifespan and healthspan. Aging (Albany NY). 2018; 10:573–91. 10.18632/aging.101414 29676998 PMC5940111

