# Correction for: Accelerated DNA methylation age and the use of antihypertensive medication among older adults

**DOI:** 10.18632/aging.101980

**Published:** 2019-05-15

**Authors:** Xu Gao, Elena Colicino, Jincheng Shen, Allan C. Just, Jamaji C. Nwanaji-Enwerem, Brent Coull, Xihong Lin, Pantel Vokonas, Yinan Zheng, Lifang Hou, Joel Schwartz, Andrea A. Baccarelli

**Affiliations:** 1 Department of Environmental Health Sciences, Mailman School of Public Health, Columbia University, New York, NY 10032, USA; 2 Department of Environmental Medicine and Public Health, Icahn School of Medicine at Mount Sinai, New York, NY 10029, USA; 3 Department of Population Health Sciences, University of Utah, School of Medicine, Salt Lake City, UT 84132, USA; 4 Department of Environmental Health, Harvard T.H. Chan School of Public Health, Boston, MA 02115, USA; 5 Department of Biostatistics, Harvard T.H. Chan School of Public Health, Boston, MA 02115, USA; 6 Veterans Affairs Normative Aging Study, Veterans Affairs Boston Healthcare System, Department of Medicine, Boston University School of Medicine, Boston, MA 02118, USA; 7 Department of Preventive Medicine, Feinberg School of Medicine, Northwestern University, Chicago, IL 60611, USA

Original article: Aging (Albany NY) 2018; 10:3210–3228

**This article has been corrected:** The authors requested to add **Dr. Cuicui Wang** to the list of authors. **Cuicui Wang** was omitted in this publication by mistake. The correct authors' list is provided below. The authors declare that this correction does not change the results or conclusions of this paper. The authors sincerely apologize for this error.

Xu Gao^1^, Elena Colicino^2^, Jincheng Shen^3^, Allan C. Just^2^, Jamaji C. Nwanaji-Enwerem^4^, Cuicui Wang^4^, Brent Coull^5^, Xihong Lin^5^, Pantel Vokonas^6^, Yinan Zheng^7^, Lifang Hou^7^, Joel Schwartz^4^, Andrea A. Baccarelli^1^

^1^Department of Environmental Health Sciences, Mailman School of Public Health, Columbia University, New York, NY 10032, USA

^2^Department of Environmental Medicine and Public Health, Icahn School of Medicine at Mount Sinai, New York, NY 10029, USA

^3^Department of Population Health Sciences, University of Utah, School of Medicine, Salt Lake City, UT 84132, USA

^4^Department of Environmental Health, Harvard T.H. Chan School of Public Health, Boston, MA 02115, USA

^5^Department of Biostatistics, Harvard T.H. Chan School of Public Health, Boston, MA 02115, USA

^6^Veterans Affairs Normative Aging Study, Veterans Affairs Boston Healthcare System, Department of Medicine, Boston University School of Medicine, Boston, MA 02118, USA

^7^Department of Preventive Medicine, Feinberg School of Medicine, Northwestern University, Chicago, IL 60611, USA

