# Supplementary material for: Accelerated DNA methylation age and the use of antihypertensive medication among older adults
Source: Aging (Albany NY). 2018 Nov 10;10(11):3210–28. doi: 10.18632/aging.101626 (PMC6286862; doi:10.18632/aging.101626)
Supplement: Supplementary Table S1 [file aging-10-101626-s001.pdf]

Table S1. Associations of hypertension, diabetes and metabolic syndrome with DNA methylation age, age acceleration and change rate of age acceleration [a].

	Health outcomes	Status at first visit	N	Coefficients (SE)	p-value	Status at second visit	N	Coefficients (SE)	p-value
Age acceleration	Hypertension	No	172	Ref		No	135	Ref	
		Yes	374	0.041 (0.543)	0.940	Yes	411	0.326 (0.703)	0.644
	Diabetes	No	479	Ref		No	457	Ref	
		Yes	67	0.783 (0.728)	0.283	Yes	89	0.431 (0.804)	0.592
	Metabolic syndrome	No	265	Ref		No	249	Ref	
		Yes	281	0.867 (0.527)	0.101	Yes	297	0.847 (0.644)	0.683
Change rate of age acceleration	Hypertension	No	172	Ref		No	135	Ref	
		Yes	374	0.144 (0.129)	0.267	Yes	411	0.219 (0.137)	0.111
	Diabetes	No	479	Ref		No	457	Ref	
		Yes	67	0.419 (0.174)	0.016	Yes	89	0.361 (0.156)	0.021
	Metabolic syndrome	No	265	Ref		No	249	Ref	
		Yes	281	0.171 (0.126)	0.176	Yes	297	0.343 (0.124)	0.061

a: Model adjusted for corresponding covariates at each visit: age, BMI, alcohol consumption, smoking, years of education, physical activity, leucocyte distribution (Houseman algorithm) and the random batch effect of DNA methylation measurement. Age acceleration was additionally adjusted for in the model for the change rate of age acceleration.
