# Supplementary material for: Accelerated DNA methylation age and the use of antihypertensive medication among older adults
Source: Aging (Albany NY). 2018 Nov 10;10(11):3210–28. doi: 10.18632/aging.101626 (PMC6286862; doi:10.18632/aging.101626)
Supplement: Supplementary Table S2 [file aging-10-101626-s002.pdf]

Table S2. Associations of clinical biomarkers with DNA methylation age, age accelerations and change rate of age acceleration[a].

	Biomarkers	First visit		Second visit	
		Coefficients (SE)	p-value	Coefficients (SE)	p-value
Age acceleration	SBP	9.5 e-3 (0.014)	0.498	-2.6 e-3 (0.016)	0.874
	DBP	-0.011 (0.027)	0.690	0.012 (0.031)	0.706
	Fasting glucose	0.017(0.009)	0.058	0.005 (0.014)	0.713
	Total cholesterol	-2.6 e-3(6.6 e-3)	0.698	-8.8 e-3 (7.5 e-3)	0.243
	HDL	-0.020 (0.019)	0.287	-0.037 (0.023)	0.100
	Triglyceride	4.1 e-4 (3.4 e-3)	0.904	-6.2 e-3 (5.1 e-3)	0.227
	Uric acid	0.084 (0.168)	0.617	-0.113 (0.205)	0.584
	Calcium	0.665 (0.648)	0.306	-0.273 (0.823)	0.740
	Phosphorus	-0.251 (0.545)	0.645	-0.159 (0.625)	0.799
	Potassium	2.1 e-4 (2.2 e-4)	0.338	-1.0 e-4 (2.4 e-4)	0.684
	Zinc	-0.024 (0.014)	0.100	0.010 (0.016)	0.527
	Magnesium	8.1 e-5 (1.9 e-3)	0.966	-7.9 e-4 (2.1 e-3)	0.706
	Sodium	7.2 e-5 (1.3 e-4)	0.587	-1.4 e-4 (1.6 e-4)	0.364
Change rate of age acceleration	SBP	2.4 e-3 (3.4 e-3)	0.477	4.1 e-3 (3.6 e-3)	0.260
	DBP	1.9 e-3 (6.6 e-3)	0.773	0.011 (0.007)	0.102
	Fasting glucose	-4.0 e-3 (2.2 e-3)	0.067	-9.8 e-4 (3.0 e-3)	0.746
	Total cholesterol	2.4 e-3 (1.6 e-3)	0.146	2.3 e-3 (1.7 e-3)	0.175
	HDL	1.5 e-3 (4.6 e-3)	0.745	2.9 e-3 (5.0 e-3)	0.566
	Triglyceride	5.0 e-4 (8.3 e-4)	0.552	-9.1 e-4 (1.1 e-3)	0.423
	Uric acid	-4.2 e-3 (0.041)	0.919	-0.049 (0.048)	0.307
	Calcium	0.116 (0.160)	0.469	-0.078 (0.190)	0.684
	Phosphorus	-0.181 (0.134)	0.178	-0.106 (0.144)	0.462
	Potassium	1.1 e-5 (5.4 e-5)	0.845	-5.0 e-5 (5.2 e-5)	0.353
	Zinc	6.0 e-3 (3.6 e-3)	0.092	3.8 e-3 (3.6 e-3)	0.286
	Magnesium	1.7 e-4 (4.7 e-4)	0.715	-6.1 e-4 (4.6 e-4)	0.184
	Sodium	-9.5 e-6 (3.3 e-5)	0.771	-7.0 e-5 (3.4 e-5)	0.055

a: Model adjusted for corresponding covariates at each visit: age, BMI, alcohol, smoking, years of education, physical activity and leucocyte distribution (Houseman algorithm). Age acceleration was additionally adjusted for in the model for the change rate of age acceleration.
