# Supplementary material for: Accelerated DNA methylation age and the use of antihypertensive medication among older adults
Source: Aging (Albany NY). 2018 Nov 10;10(11):3210–28. doi: 10.18632/aging.101626 (PMC6286862; doi:10.18632/aging.101626)
Supplement: Supplementary Table S3 [file aging-10-101626-s003.pdf]

Table S3. Sensitivity analyses: associations of any antihypertensive medication use with age acceleration and change rate of age acceleration for Hannum DNA methylation age and DNA methylation Phenotypic age.

a. Cross-sectional associations with age acceleration and change rate of age acceleration.

Visit	Types of aging biomarkers	Use of any antihypertension medication	N	Age acceleration				Change rate of age acceleration			
				Model 1 [a]		Model 2 [b]		Model 1		Model 2	
				Coefficients (SE)	p-value	Coefficients (SE)	p-value	Coefficients (SE)	p-value	Coefficients (SE)	p-value
**First visit**	DNA methylation age (Hannum)	No	233	Ref		Ref		Ref		Ref	
		Yes	313	0.899 (0.406)	0.014	2.150 (0.727)	0.003	0.050 (0.073)	0.489	0.137 (0.112)	0.383
	DNA methylation Phenotypic age	No	233	Ref		Ref		Ref		Ref	
		Yes	313	1.406 (0.568)	0.0001	1.687 (0.999)	0.002	0.038 (0.108)	0.731	0.037 (0.198)	0.854
**Second visit**	DNA methylation age (Hannum)	No	163	Ref		Ref		Ref		Ref	
		Yes	383	0.925 (0.445)	0.113	1.801 (0.992)	0.153	0.016 (0.075)	0.832	0.015 (0.153)	0.920
	DNA methylation Phenotypic age	No	163	Ref		Ref		Ref		Ref	
		Yes	383	1.732 (0.573)	0.003	1.692 (1.340)	0.087	0.091 (0.116)	0.435	0.221 (0.238)	0.355

a: Model 1: Adjusted for covariates at each visit: age + leukocyte distribution (Houseman algorithm) + random effect (batch effect of DNA methylation measurement). Age acceleration was additionally adjusted for in the model for the change rate of age acceleration;

b: Model 2: Model 1 + BMI + smoking status + alcohol consumption + physical activity + years of education + total cholesterol + HDL + triglycerides + fasting glucose + SBP + hypertension + stroke + CHD + diabetes + cancer.

b. Longitudinal associations with change rate of age acceleration.

Types of aging biomarkers	Change of any antihypertension medication use	N	Change rate of age acceleration			
			Model 1 [a]		Model 2 [b]	
			Coefficients (SE)	p-value	Coefficients (SE)	p-value
DNA methylation age (Hannum)	Never used	150	Ref		Ref	
	Stopped use after first visit	13	-0.789 (0.241)	0.001	-0.861 (0.287)	0.003
	Started use after first visit	83	0.055 (0.109)	0.614	0.039 (0.123)	0.753
	Continuous use	300	0.139 (0.112)	0.187	0.151 (0.148)	0.199
DNA methylation Phenotypic age	Never used	150	Ref		Ref	
	Stopped use after first visit	13	-0.516 (0.355)	0.147	-0.540 (0.412)	0.191
	Started use after first visit	83	0.159 (0.162)	0.325	0.116 (0.178)	0.517
	Continuous use	300	0.187 (0.124)	0.227	0.146 (0.215)	0.130

a: Model 1: Adjusted for: age (first visit) + age acceleration (first visit) + leukocyte distribution (first visit, Houseman algorithm) + random effect (batch effect of methylation measurement at first visit);

b: Model 2: Model 1 + BMI (first visit) + smoking status (first visit) + alcohol consumption (first visit) + physical activity (first visit) + years of education (first visit) + total cholesterol (first visit) + HDL (first visit) + triglycerides (first visit) + fasting glucose (first visit) + SBP (first visit) + hypertension (first visit) + stroke (first visit) + CHD (first visit) + diabetes (first visit) + cancer (first visit).
