# Supplementary material for: Accelerated DNA methylation age and the use of antihypertensive medication among older adults
Source: Aging (Albany NY). 2018 Nov 10;10(11):3210–28. doi: 10.18632/aging.101626 (PMC6286862; doi:10.18632/aging.101626)
Supplement: Supplementary Figure S1 [file aging-10-101626-s004.pdf]

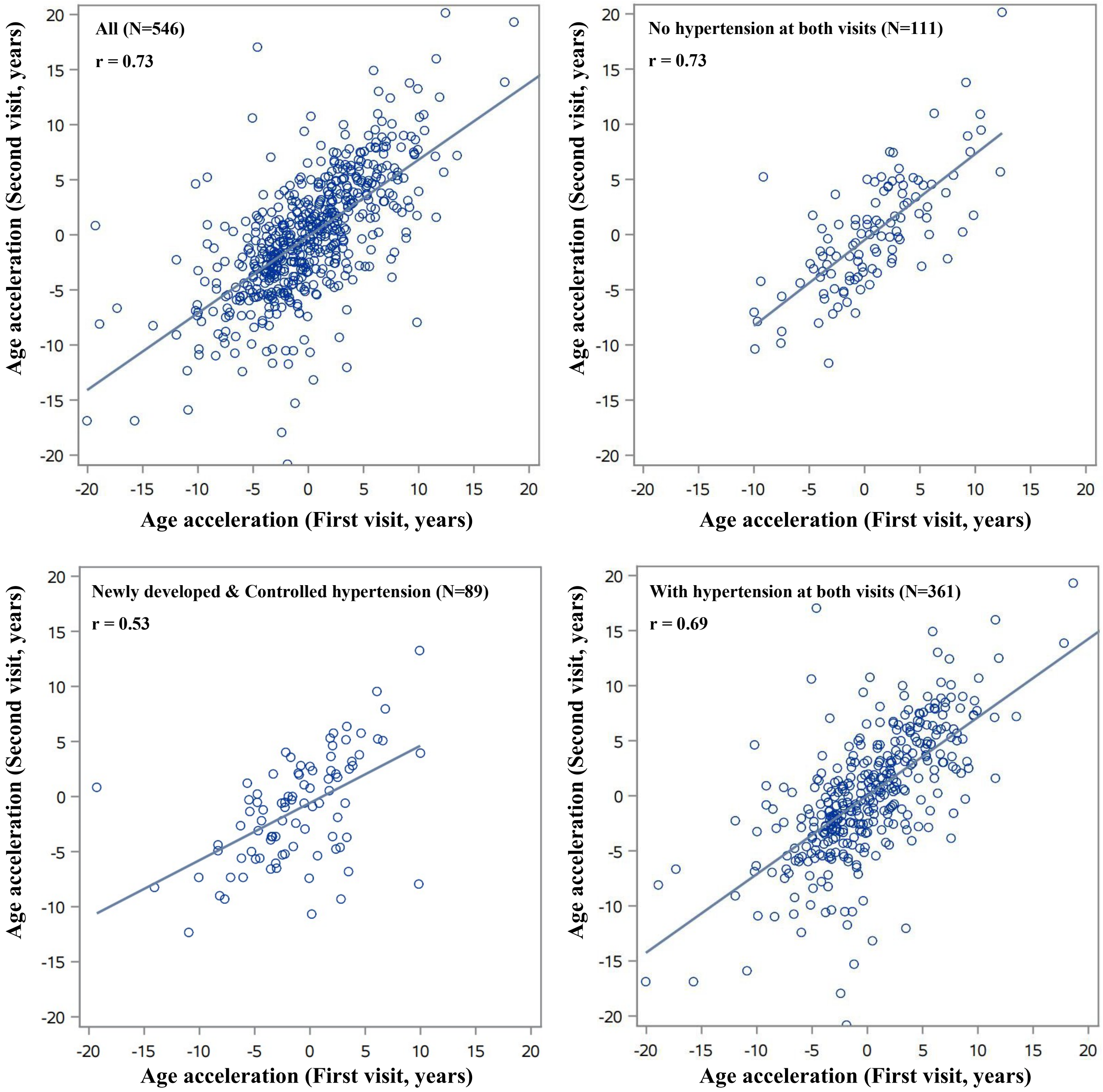

Figure S1. Correlations of age accelerations between the first and second visit based on the status of hypertension at each visit.
